# Soft X-ray characterization of halide perovskite film by scanning transmission X-ray microscopy

**DOI:** 10.1038/s41598-022-08256-3

**Published:** 2022-03-16

**Authors:** Haeyeon Jun, Hee Ryung Lee, Denis Tondelier, Bernard Geffroy, Philip Schulz, Jean-Éric Bourée, Yvan Bonnassieux, Sufal Swaraj

**Affiliations:** 1 Synchrotron SOLEIL, L’Orme Des Merisiers Saint-Aubin, BP 48, 91192 Gif-sur-Yvette Cedex, France; 2 CEA, CNRS, NIMBE, LICSEN, Université Paris-Saclay, 91191 Gif-sur-Yvette, France; 3 LPICM, CNRS, Ecole Polytechnique, Institut Polytechnique de Paris, Route de Saclay, 91128 Palaiseau, France; 4 CNRS, Institut Photovoltaïque d’Île de France (IPVF), UMR 9006, 18, Boulevard Thomas Gobert, 91120 Palaiseau, France

**Keywords:** Chemistry, Materials science, Nanoscience and technology

## Abstract

Organic–inorganic metal halide perovskites (MHPs) have recently been receiving a lot of attention due to their newfound application in optoelectronic devices, including perovskite solar cells (PSCs) which have reached power conversion efficiencies as high as 25.5%. However, the fundamental mechanisms in PSCs, including the correlation of degradation with the excellent optoelectrical properties of the perovskite absorbers, are poorly understood. In this paper, we have explored synchrotron-based soft X-ray characterization as an effective technique for the compositional analysis of MHP thin films. Most synchrotron-based studies used for investigating MHPs so far are based on hard X-rays (5–10 keV) which include various absorption edges (Pb L-edge, I L-edge, Br K-edge, etc.) but are not suited for the analysis of the organic component in these materials. In order to be sensitive to a maximum number of elements, we have employed soft X-ray-based scanning transmission X-ray microscopy (STXM) as a spectro-microscopy technique for the characterization of MHPs. We examined its sensitivity to iodine and organic components, aging, or oxidation by-products in MHPs to make sure that our suggested method is suitable for studying MHPs. Furthermore, methylammonium triiodide with different deposition ratios of PbI_2_ and CH_3_NH_3_I (MAI), and different thicknesses, were characterized for chemical inhomogeneity at the nanoscale by STXM. Through these measurements, we demonstrate that STXM is very sensitive to chemical composition and homogeneity in MHPs. Thus, we highlight the utility of STXM for an in-depth analysis of physical and chemical phenomena in PSCs.

## Introduction

Organic–inorganic metal halide perovskites (MHPs) with ABX_3_ structure have been regarded as promising materials for various optoelectronic applications over the past few years. MHPs have remarkable optoelectronic properties, including a tunable bandgap, long charge carrier lifetimes, a small exciton binding energy, and a high absorption coefficient^1–3^. These properties are well suited for electrical and optical applications such as photovoltaics, light-emitting diodes, resistive-switching memory devices, and field-effect transistors^4–7^. Among various applications, perovskite-based solar cells (PSCs) conspicuously have achieved a certified power conversion efficiency (PCE) of 25.5% within ten years of development, comparable to that of monocrystalline silicon based solar cells^8^.

However, one of the challenging issues that hinders successful commercialization of PSCs is still low operational stability. The mechanisms of degradation in a bulk perovskite layer and interfaces between perovskite and transport layers are caused by various factors of the operational environment such as oxygen, moisture, heat, light, and electrical field. To improve the stability, many researchers have tried to investigate them by different techniques and have observed the degradation phenomena under various conditions^9–11^. However, understanding of the intrinsic degradation attributed to light, heat, and electrical fields still remains vague due to the complexity of these materials. For a better understanding of the detailed mechanisms of the phenomena, advanced analytical techniques are needed, which enable investigation of nanoscale chemical composition with high sensitivity for every element. Combining spatially resolved chemical analysis with techniques probing material’s local opotoelectronic properties is one way to understand the link between composition and performance of PSCs.

Since the advent of PSCs, characterizations based on synchrotron radiation have been employed to deepen our understanding of the correlation between non-stoichiometry and halide migration. Synchrotron radiation sources can produce highly collimated monochromatic X-rays with high flux. Owing to these beam characteristics, structural, chemical, and physical properties can be investigated across multiple length scales^12^. In many instances, there have been applications of synchrotron radiation-based characterization, which has provided decisive answers to the physico-chemical mechanisms in MHPs that cannot be revealed by laboratory-based characterization. Adhyaksa et al.^13^ employed synchrotron-based nanoprobe X-ray diffraction (nano-XRD) to examine the effect of grain-boundaries in MHP and verified that the disordered region at grain boundaries is amorphous giving rise to locally bright photoluminescence intensity, which is quite an anomalous grain boundary behavior. Li et al.^14^ also used nano-XRD to study the influence of nanoscale residual strains on CsPbBr_3_ thin film on the stability of perovskite crystal structure and photoluminescence (PL). Luo et al.^15^ could observe nanoscale heterogeneous distribution of chlorine/iodine mass ratio in MAPb(I_1−*x*_Cl_*x*_)_3_ according to the method of synthesis and deposition using synchrotron-based nano X-ray fluorescence (XRF) microscopy. With the same technique they observed in CH_3_NH_3_PbBr_3_ an increase of local bromine/lead atomic ratio under an applied electric field correlated with a local increase of photoluminescence. Furthermore, Stuckelberger et al. carried out nano-XRF with X-ray beam-induced current (XBIC) on mesoscopic MAPbI_3_ solar cells. The combined XRF/XBIC measurements give valuable correlation between elemental distributions and charge collection efficiency^16^. Philippe et al.^17^ demonstrated the depth-dependent distribution of Cs and Rb and the role of the distributions in mixed-cation RbCsMAFA perovskite with hard X-ray photoelectron spectroscopy (HAXPES). In addition, Sekimoto et al.^18^ performed HAXPES to investigate the effect of hole transport layer (HTL) on light-induced degradation and assessed the accumulation of iodine and lead at the interface between perovskite film and HTL.

**Figure 1:**
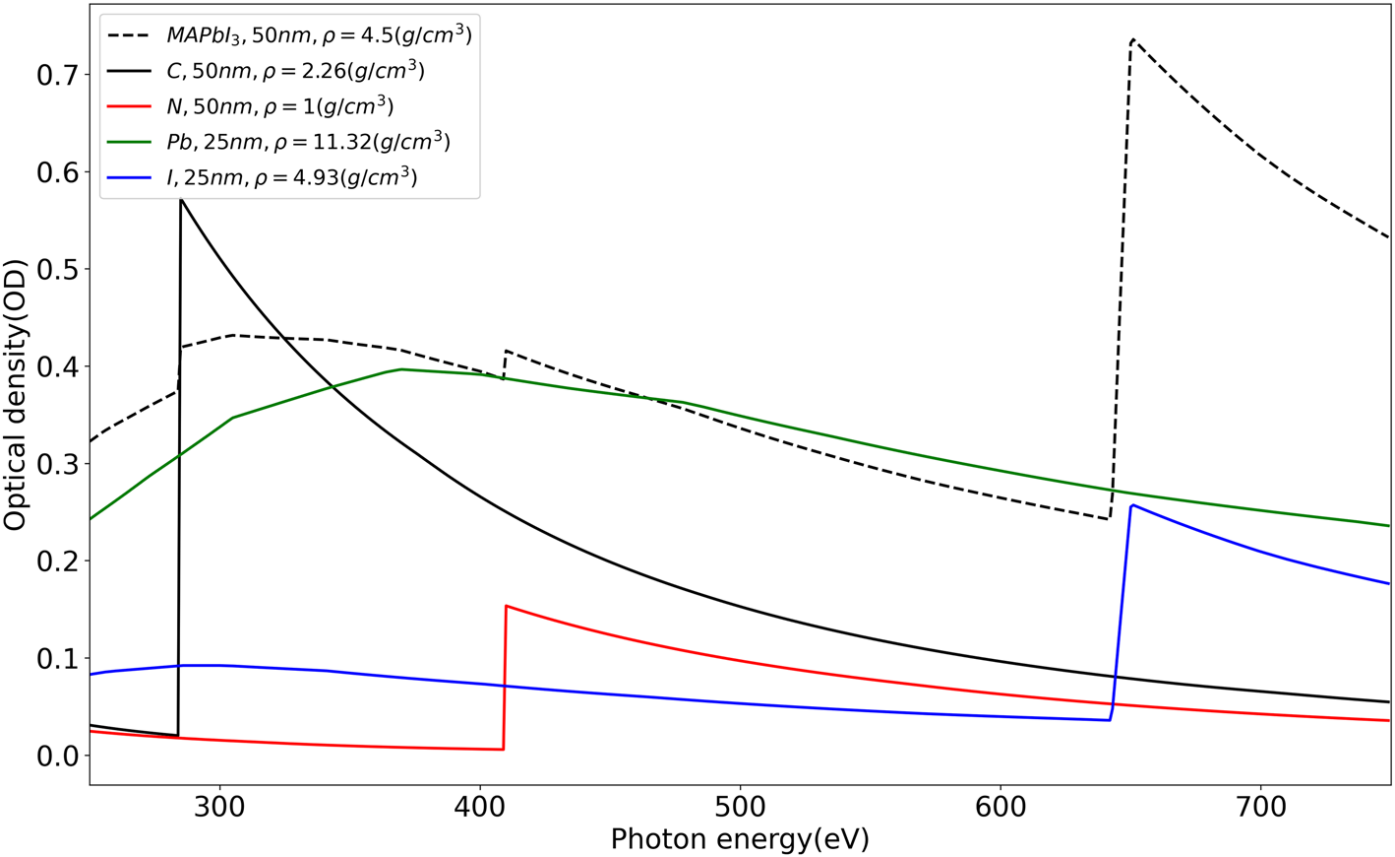
Simulations for X-ray absorption spectrum of MAPbI_3_ (top) and the different perovskite elements including C, N, Pb and I (bottom) in soft X-ray region using aXis2000^27^.

The majority of these synchrotron-based studies of MHPs have employed hard X-rays in the range 5–10 keV primarily because the interesting X-ray absorption edges related to some of the elements (Pb L-edge, I L-edge, Br K-edge, etc.) in MHP compounds lie in the hard X-ray regime^17,19–21^. On the other hand, the soft X-ray regime does include the inner shell X-ray absorption edges of carbon and nitrogen (C K-edge and N K-edge) and the outer shell X-ray absorption edges of Pb and I (Pb N-edge and I M-edge) in the same regime contributing in the form of a strong background (Fig. 1). While soft X-rays remain challengingfor the investigation of MHP compunds, it does provide the opportunity to investigate the organic component of MHPs in conjunction with the lead halide component.

In principle, this energy range could be used to investigate changes in the spectroscopic fine structure of the C K-edge at 284 eV and the N K-edge at 405.5 eV, to explore the chemical changes at interfaces between perovskite active layers and charge transport layers^22^ as well as ion migration in the perovskite film itself^23^. We would like to point out that the I M absorption edge as tabulated in the Henke database^24^ was found to be 20 eV below (619 eV) that found in our studies (~ 640 eV). As pointed out by Hansen^25^ and Comes et al.^26^, the tabulated values need further investigation. In order to overcome this discrepancy, we have blue-shifted the iodine M-edge tabulated value by 20 eV in the range of (620–700 eV) before simulating the spectra using the software aXis2000^27^. The simulation parameters used are mentioned in the Fig. 1.

**Figure 2:**
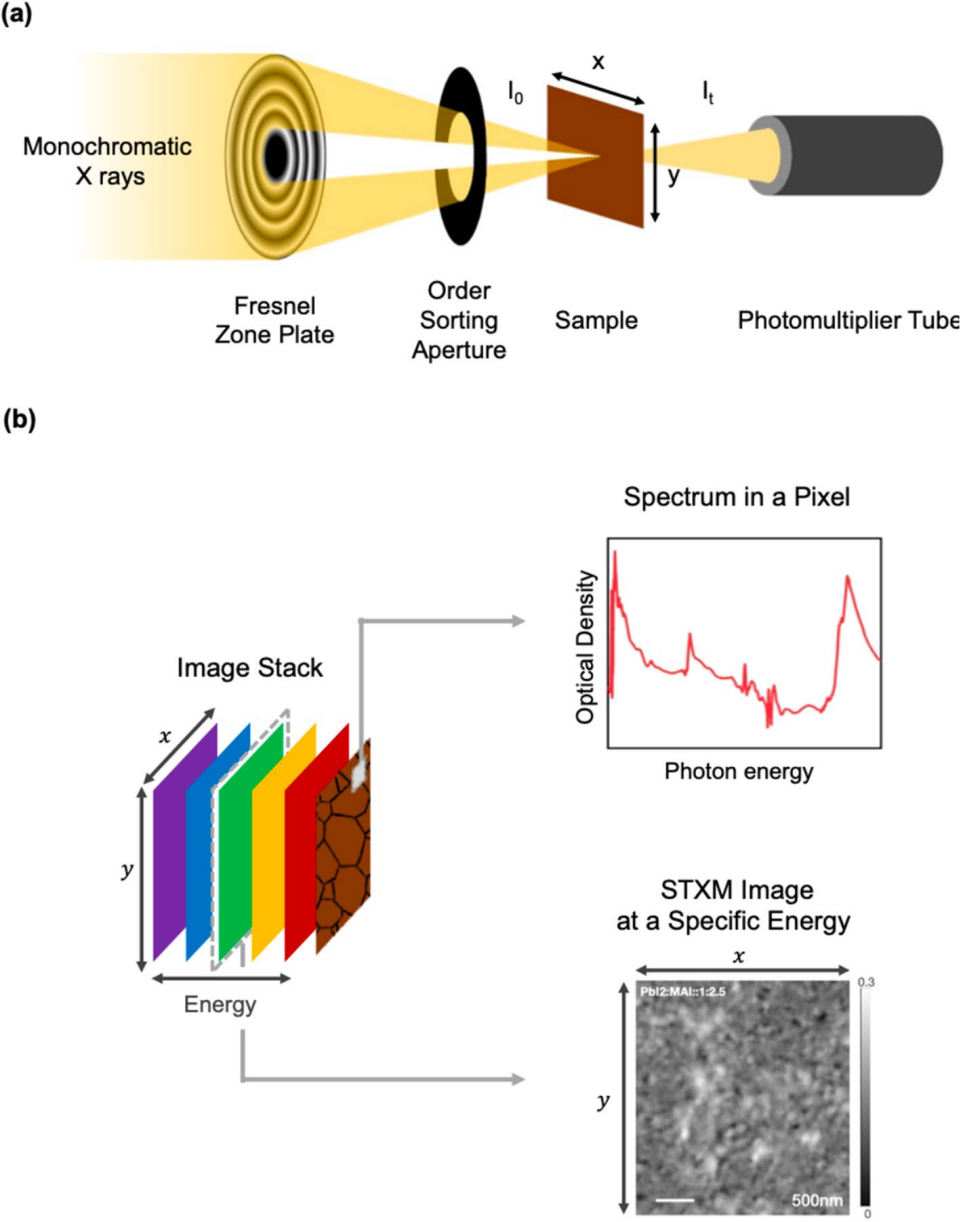
(**a**) Experimental set-up for scanning transmission X-ray microscopy (STXM). (**b**) Illustration of data acquired by STXM, including spatially resolved spectra (spectrum in a particular pixel) and STXM image at a specific energy.

In this paper, we have used scanning transmission X-ray microscopy (STXM) measurements in the soft X-ray regime (270–800 eV) to demonstrate the possibility of spectroscopy as well as microscopy using soft X-rays (soft X-ray spectro-microscopy). STXM is an experimental technique (see Fig. 2 for schematics) based on X-ray absorption spectroscopy (XAS). In most of the STXM studies the “fine structure” near the absorption edge is specifically exploited. This is referred as near edge X-ray absorption fine structure (NEXAFS) spectroscopy or X-ray absorption fine structure (XAFS). In XAS, the absorption spectra are usually represented in terms of optical density (OD) given as $ln\left( {\frac{{I_{0} }}{I}} \right)$ where *I*_*o*_ is the incident photon flux and *I* is transmitted photon flux. $I$ and *I*_*o*_ (and hence OD) are correlated to the mass absorption coefficient $\left( {\mu \left( E \right)} \right)$, density ($\rho$), and thickness (t) from the Beer-Lamber law as shown in Eq. (1).

1 $$I = I_{0} e^{{\left( { - \mu \left( E \right)\rho t} \right)}}$$

2 $$ln\left( {\frac{{I_{0} }}{I}} \right) = OD = \mu \left( E \right)\rho t$$

This spectro-microscopy technique allows us to obtain quantitative chemical composition maps of the bulk of materials with a high spatial resolution (~ 30 nm in this study).

In order to demonstrate the utility of soft X-ray STXM for the characterization of MHPs, we have verified its sensitivity to (a) various elements/components that are contained in MHPs and (b) various aging or oxidation by-products. To achieve the above objectives, the following samples were prepared: methylammonium lead iodide (CH_3_NH_3_PbI_3_, MAPbI_3_), lead iodide (PbI_2_), methylammonium iodide (CH_3_NH_3_I, MAI), lead chloride (PbCl_2_), potassium iodide (KI), and lead oxide (PbO). PbI_2_ and MAI are major components of the MHP, MAPbI_3_. PbCl_2_ and KI films are used as a comparison standard to test whether STXM is sensitive to C, N, Pb and I in this energy range. The X-ray absorption spectrum of PbO obtained from STXM is measured to identify the by-product issued from the degradation of MAPbI_3_ (MHP in this study). Furthermore, MAPbI_3_ samples with different PbI_2_:MAI ratios and of various thicknesses were prepared and compared to highlight the chemical and thickness sensitivity of STXM.

## Results and discussions

### Identification of components and by-products

**Figure 3:**
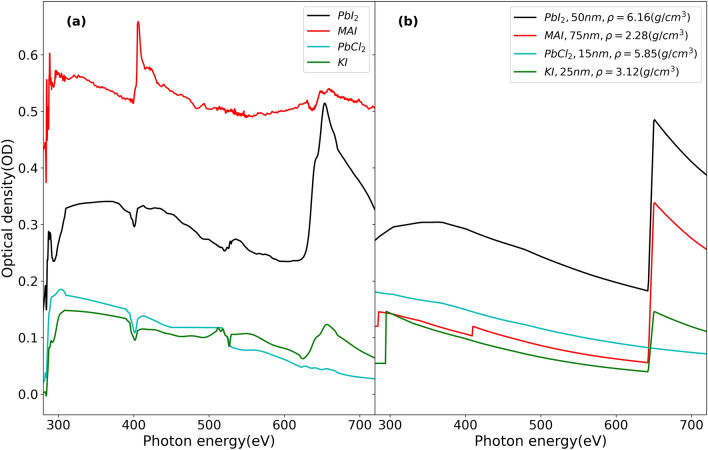
(**a**) Experimental XAS spectra (**b**) Simulated spectra^27^ of PbI_2_, MAI, PbCl_2_ and KI in the range of 280–720 eV. Simulation parameters are mentioned in legend of (**b**).

Figure 3a shows NEXAFS spectra of PbI_2_, MAI, PbCl_2_, and KI, covering the C K-edge, N K-edge, O K-edge, I M-edge, and Pb N-edge along with the corresponding simulated spectra (Fig. 3b) of appropriate thickness. In the spectrum of PbI_2_, a broad Pb N-edge in the form of a ‘bump’ can be observed at around 370 eV. A strong I M-edge at 631 eV is also observed. The spectrum of MAI presents three edges at ~ 285 eV, ~ 395 eV and ~ 631 eV that correspond to the C K-edge, N K-edge and I M-edge, respectively. In the case of KI, the I M-edge at ~ 631 eV and K L-edge at ~ 295 eV are observed in the spectra. The spectra of PbCl_2_ shows a trend similar to the simulated spectrum but presents variations at C, N, and O edge energy positions that most likely correspond to contamination or incorrect normalization. In comparison to the simulated spectrum, the experimental MAI spectrum appears with an inhibited I M-edge. This fact is supported by energy-dispersive X-ray spectroscopy (EDX) analysis of MAI films (Fig. S1 and Table S1), wherein the iodine atomic percent was observed to be small compared with those of carbon and nitrogen. An offset in the OD scale can also be observed in the case of MAI spectra compared to the simulated spectra. We speculate that this could be due to either contamination of the MAI film or radiation-related damage. This would be investigated in further studies.

**Figure 4:**
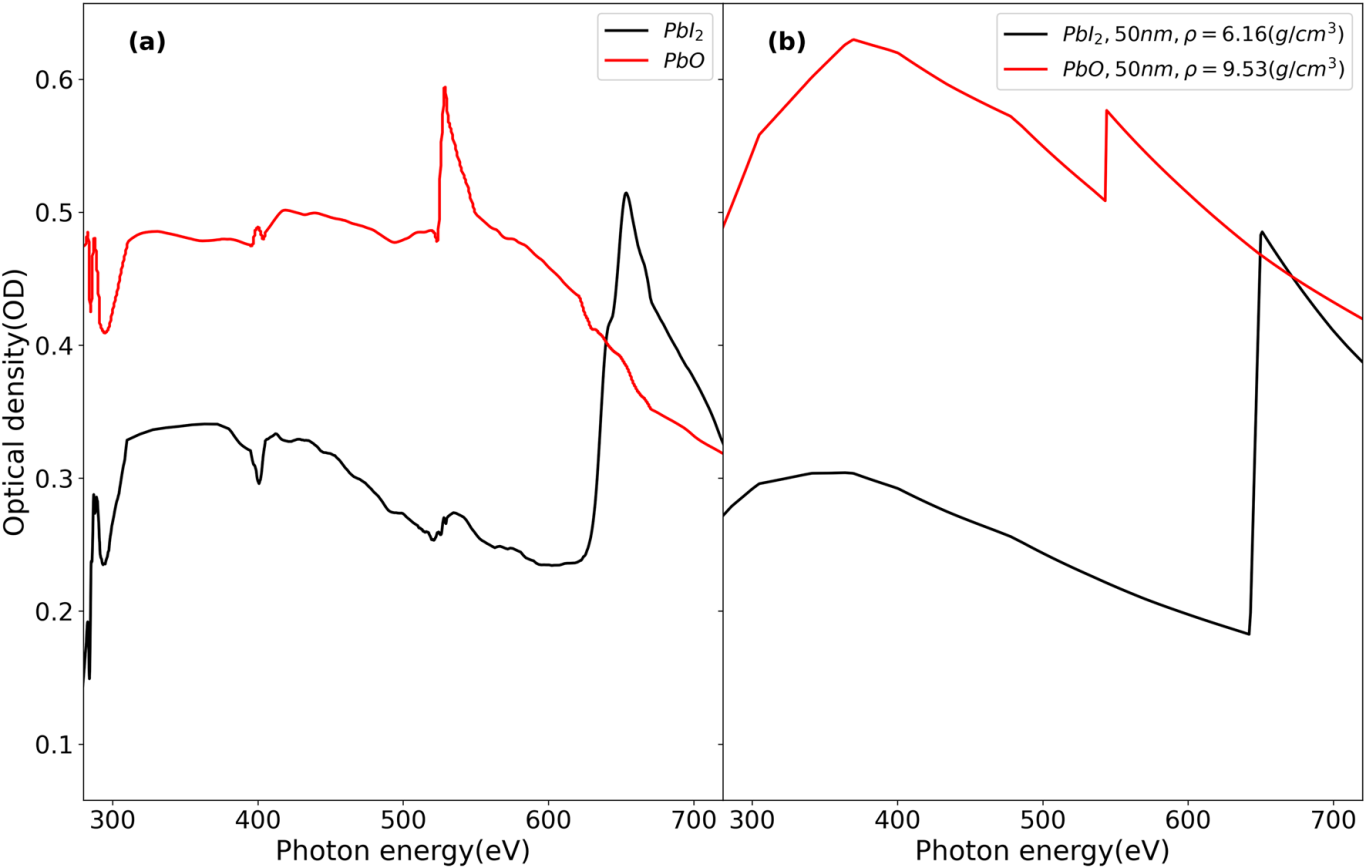
(**a**) Experimental NEXAFS spectra and (**b**) Simulated spectra^27^ of PbI_2_, and PbO in the range of 280–720 eV. Simulation parameters are mentioned in legend of (**b**).

In order to investigate physical and chemical phenomena in PSCs using STXM, we need to identify not only the components of the perovskite but also potential by-products from the film processing and degradation processes. For example, in the study of stability in MAPbI_3_, the major by-products of aged perovskite are PbI_2_ and PbO^28–32^. Their corresponding XAS spectra are shown in Fig. 4. We confirm that PbI_2_ and PbO have broad Pb N-edge at 370 eV in common. However, the I M-edge only exists in the OD spectrum of PbI_2_, whereas the PbO spectrum is relatively flat at 370 eV. Furthermore, in the OD spectrum of PbO, we find a prominent O K-edge at 543 eV. Presence of oxygen moieties can also be detected in the PbI_2_ spectra, highlighting the sensitivity of these techniques to degradation by products of aging in air. These two results in Figs. 3 and 4 confirm that each component of MHPs such as MAI and PbI_2_ can be detected and distinguished from other components. The absorption spectra of KI (pure I, no Pb) and PbCl_2_ (only Pb, no I) in Fig. 3 show that we are able to reliably attribute I and Pb contributions to spectra of films that contain both elements. Similarly, the absorption spectra of PbI_2_ and PbO presented in Fig. 4 are compared to their simulated spectra of appropriate thickness to assure the reliable detection of these by-products of aged MAPbI_3_ in soft X-ray region. The low energy anomaly observed in PbO in the C K-edge region (280–310 eV) in Fig. 4a could be due to bad normalization or beamline optics contamination.

### Characterization of MAPbI_3_

**Figure 5:**
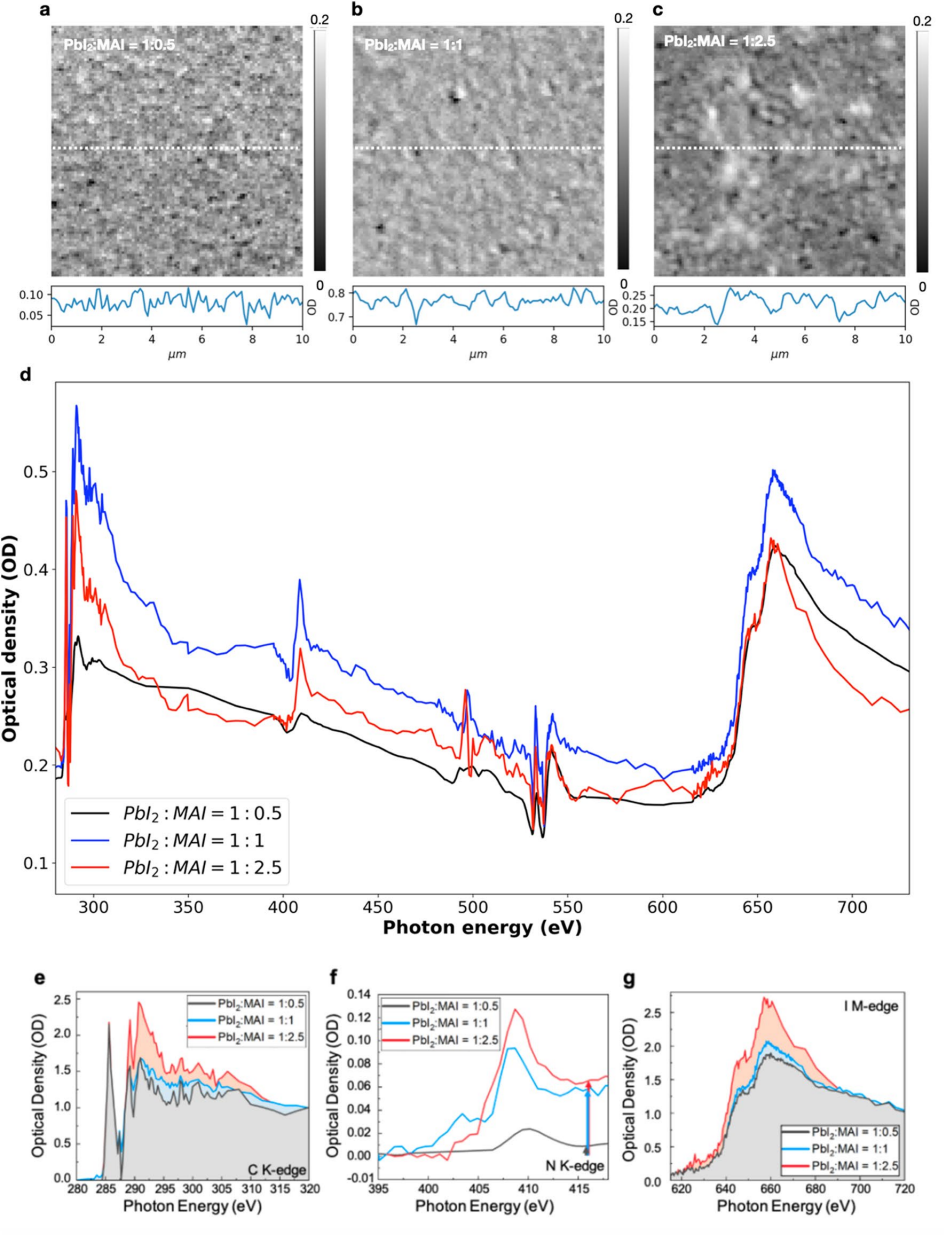
STXM images and spectra of MAPbI_3_ with different PbI_2_ to MAI ratios. STXM images and line profile (taken from white dotted line in the image) at 662 eV with the ratio of (**a**) 1:0.5 (**b**) 1:1 (**c**) 1:2.5, (**d**) NEXAFS spectra in the entire energy region (280–730 eV), (**e**) edge normalized C K-edge, (**f**)N K-edge, and (**g**) I M-edge region of MAPbI_3_ with different PbI_2_ to MAI ratios. The shaded regions in (**e**) and (**g**) indicate the integrated C K-edge spectra and I M-edge spectra, and the arrows in (**f**) represents the N K-edge jumps.

In general, MAPbI_3_, one of MHPs, is formed by the reaction of PbI_2_ and MAI. The large grain size with grain boundaries in MAPbI_3_ are generally considered to result in high photovoltaic performance with low recombination rate^33,34^. The relative ratio of PbI_2_ to MAI in vacuum deposition process considerably affects the grain size in MAPbI_3_. Figure 5 illustrates the use of STXM to characterize MAPbI_3_ with different PbI_2_ to MAI ratios. STXM images of MAPbI_3_ with PbI_2_ to MAI ratio of 1:0.5, 1:1 and 1:2.5 at 662 eV are shown in Fig. 5a–c, respectively. A horizontal line profile taken from the center of the image in each case is also presented. Similar to other studies^35,36^, we observe that as the amount of MAI increases, the grain size increases. The increase in grain size can be interpreted from the line profiles and also from Fig. 5a–c by the increase in domain size (dimension of regions of similar intensity) and decrease in image “roughness”. Figure 5d indicates spectra of MAPbI_3_ with the different ratios that cover, C K edge, N K-edge, O K-edge, I M-edge and Pb N-edge energy range. The overall difference in the OD of the three spectra, especially that with the PbI_2_ to MAI ratio of 1:1 arises from the difference in film thickness. In order to compare the spectra and to obtain a semi-quantitative (relative) measure of organic molecules, the integrated area under the edge-normalized C K-edge NEXAFS spectra (Fig. 5e), the edge-jump in N K-edge NEXAFS spectra (Fig. 5f) and the integrated area under the edge-normalized I M-edge NEXAFS spectra (Fig. 5e) are presented in Table 1. In each case a linear background was removed before processing. Investigations related to semi-quantitative studies using NEXAFS spectra often use edge-normalized spectra^37–40^. The integrated area under both the C K-edge spectra and the I M-edge spectra as well as the edge jump in N K-edge spectra (Table 1) can be clearly seen to be increasing for samples with higher MAI content.

**Table 1:** Integrated area under the edge-normalized C K-edge NEXAFS, the edge jump in N K-edge NEXAFS spectra and the integrated area under the edge-normalized I M-edge of various MAPbI_3_ samples.

PbI_2_: MAI deposition ratio in MAPbI_3_	Area under the edge-normalized C K-edge NEXAFS spectra (OD^2^)	Edge jump (OD) in N K-edge NEXAFS	Area under the edge-normalized I K-edge NEXAFS spectra (OD^2^)
1:0.5	35	0.008	149
1:1	39	0.058	153
1:2.5	45	0.065	169

Using STXM, we confirm that as the contents of MAI, which reacts with PbI_2_, increases, the “higher quality” of MAPbI_3_ films with larger grain size can be obtained. These results are coherent with lab-based characterization, including scanning electron microscopy (SEM) and EDX (Fig. S1 and Table S1). Here we would like to point out two major differences between EDX and STXM as characterization tools. The possibility of identifying chemical states (oxidation states, chemical bonds etc.) by STXM as compared to EDX is one of the most important aspects (discussed later for this study). In addition, while EDX measurements can be considered relatively fast, it is also well known that sample damage is much more prominent in EDX as compared to STXM measurements.

**Figure 6:**
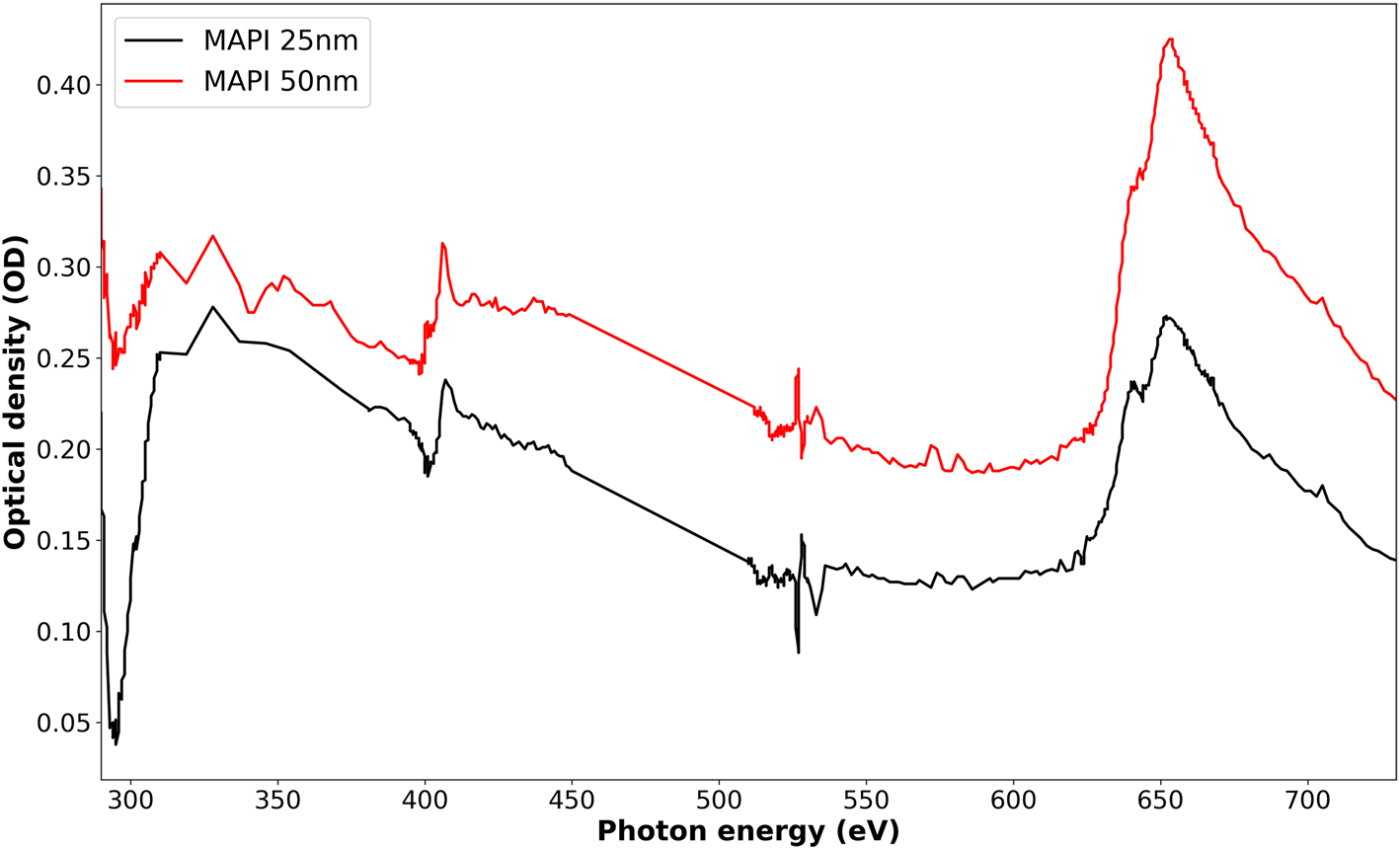
XAS spectra of MAPbI_3_ with optimized PbI_2_ to MAI ratio (1:2.5) and two different thicknesses of 25 nm and 50 nm.

Figure 6 shows XAS obtained from STXM measurements of MAPbI_3_ with optimized ratio (PbI_2_:MAI = 1:2.5) and two different thicknesses. The two spectra of MAPbI_3_ with 25 nm and 50 nm thicknesses have similar features with the expected difference in OD that corresponds to the difference in thickness. The clearly observed essential absorption edges (C K-edge, N K-edge, O K-edge and I M-edge) further confirm the reliability of STXM to detect thickness variations in MAPbI_3_ films with similar chemical composition. The sharp drop observed in the C K-edge region is possibly due to incorrect normalization in this region or due to carbon contamination on the beamline optics during these particular measurements. We do note that the OD does not show a perfect proportionality to thickness changes in Fig. 6. We suspect a larger error in measurements of lower thicknesses during deposition.

**Figure 7:**
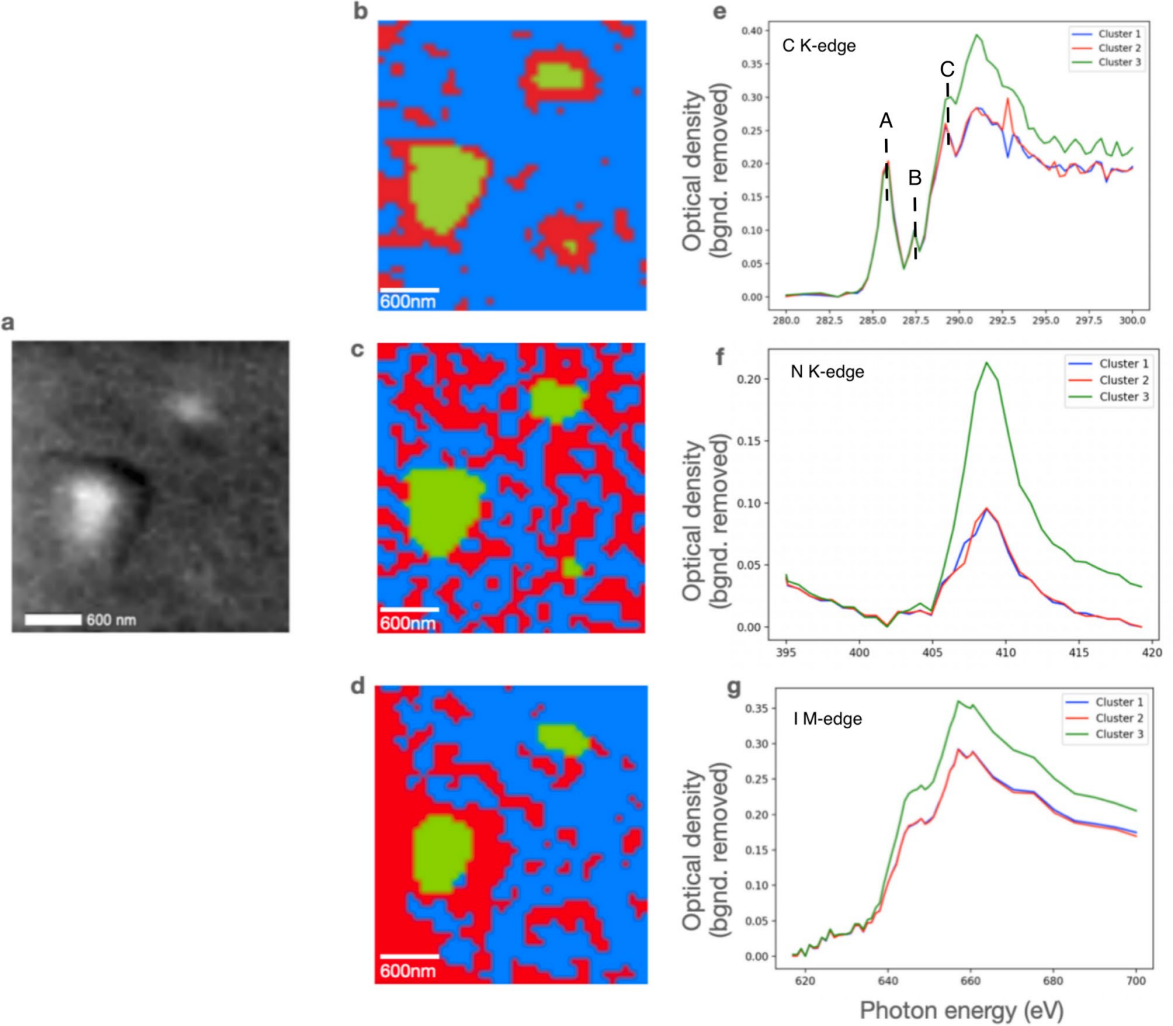
(**a**) STXM image at 662 eV of MAPbI_3_ with PbI_2_ to MAI ratio of 1:2.5. Cluster images of MAPbI_3_ in the (**b**) C K-edge, (**c**) N K-edge, and (**d**) I M-edge region, Corresponding average cluster XAS spectra of cluster analysis in the (**e**) C K-edge, (**f**) N K-edge, and (**g**) I M-edge region (Color of each spectrum corresponds to color of each cluster on the map).

Inhomogeneous morphology was observed in MAPbI_3_ with 1:2.5, and consequently a spatially resolved analysis was performed to investigate the chemical homogeneity in MAPbI_3_. Figure 7a shows a STXM image of MAPbI_3_ with the ratio of 1:2.5 at 662 eV. Figure 7b–d indicate the C, N, and I cluster maps of MAPbI_3_ for the same region obtained from cluster analysis performed by the software MANTIS^41^. Figure 7e–g are corresponding background removed XAS spectra for different clusters represented in the cluster maps. In all the cases, the spectral shape according to the clusters can be considered similar, and the variation observed arises only from the overall intensity. The intensities of spectra arising from clusters 1 and 2 are almost identical but vary in each case with the spectra of cluster 3. Considering the similarity in maps and spectra of clusters 1 and 2, the morphological and chemical homogeneities are obvious. The pre-edge spectral features A (286 eV) and B (~ 287.5 eV) in C K-edge spectra (Fig. 7e) are related to C1s → π*(C=C) and C1s → π*(C=O) transitions respectively^41^. In this first study we speculate that these features indicate the presence of oxidation and/or degradation products due to the samples being exposed to air. The feature C(~ 289.5 eV) and the shoulder just below it most likely arise from C1s → σ*(C-H)/Rydberg peak^42^. Higher energy features in C K-edge (> 290 eV) and in N K-edge (> 405 eV) correspond to C1s → σ* and N1s → σ* transitions. The sensitivity of these two absorption edges along with Oxygen K-edge (not fully utilized in this study due to beamtime constraints) to chemical moieties is well known^43^ and spectral features in these edges could be utilized for identification of various functional groups in different MHP’s.

The increase in OD of cluster 3 spectra indicates that while the chemical homogeneity persists on the grain, the thickness is higher. These cluster maps obtained from STXM provide spatially resolved spectra of not only the halide component but also organic components. They could be valuable in accessing chemical changes that could arise from aging or different kinds of stress.

## Conclusion

In this study, we highlight the utility of STXM in the soft X-ray regime to characterize organic–inorganic MHPs. Compared with hard X-ray based characterization, soft X-ray based characterization allows investigation of X-ray absorption edges of both inorganic (especially I) and organic (C and N) components.

We have presented the XAS spectra of MAI, PbI_2_, and PbCl_2_ and KI to examine the sensitivity of components in MAPbI_3_ including C, N, Pb in the soft X-ray energy range. The measured spectra accord well with the simulated spectra except for MAI. The difference between experimental and simulation spectra in MAI is caused by the film of MAI with low concentration of iodine. The fact was supported by EDX results. In addition, XAS spectra of PbO, typical by-product formed by oxidation, were measured and found to have expected features. Through these measurements, we have demonstrated that soft X-ray absorption spectra are sensitive to various chemical components MHP’s and its degradation by products.

Furthermore, taking advantage of the spatial sensitivity of STXM, MAPbI_3_ with different deposition ratios of PbI_2_ and MAI, and the thickness of MAPbI_3_ film and its homogeneity were characterized in detail. We observed that the area under the edge-normalized C K-edge XAS spectra and the edge jump in N K-edge XAS spectra increases as the amount of MAI increases. In addition, we have shown that the OD is proportional to the thickness of MAPbI_3_ with an optimized deposition ratio of 1:2.5, which follows the Beer-Lambert law. In the chemical homogeneity test, the shapes of XAS spectra in the three different nanoscale clusters were found to be the same. Changes in film thickness due to grain formation manifests itself in the form of overall increase in intensity and edge-jump. The above-mentioned measurements reveal that STXM enables study of morphological and chemical homogeneity of MHPs, on the nanometer scale.

We anticipate that STXM measurements along with further detailed data processing that includes the analysis of the near edge “fine structure” will provide detailed chemical information of halide perovskite layer and interfaces between perovskite and charge transport layers in perovskite solar cells at the nanoscale. Furthermore, in-operando STXM using particular sample environments such as electrical bias and heating, etc., will be the next obvious steps that could lead to crucial clues for the mechanism of operational degradation of perovskite solar cells.

## Experimental section/methods

### Sample preparation

All samples including PbO (Sigma Aldrich, 99%), PbCl_2_ (Alpha Aesar, ultra-dry, 99.99%), KI (Sigma Aldrich, ≥ 99%), MAI (Greatcell Solar, > 99.99%), PbI_2_ (Alpha Aesar, ultra-dry, 99.999%. and MAPbI_3_ are prepared onto a Si_3_N_4_ window (50 nm thicknesses, frame size of 5 × 5 mm^2^, Norcada Inc., Edmonton, Canada). PbO, PbCl_2_, KI and MAI are prepared by the wet process. On the other hand, PbI_2_ and MAPbI_3_ are based on the dry process. The 0.05 M of PbO was dissolved in deionized water. Each PbCl_2_ and KI solution was prepared with 0.025 g ml^−1^ concentration in *N*, *N*-Dimethylformamide (DMF, Sigma Aldrich, anhydrous, 99.8%). MAI solution was prepared by dissolving MAI into DMF with a concentration of 0.125 M. All PbO, PbCl_2_, KI, and MAI films are spin-coated at 3000 rpm in an N_2_ environment. PbI_2_ with the thickness of 50 nm was prepared by vacuum deposition. PbI_2_ was sublimated in a vacuum chamber at 300 °C, and the rate of PbI_2_ was 1 Ås^−1^. During the deposition, the vacuum level was 1.6 × 10^–6^ mbar. MAPbI_3_ of 25 nm and 50 nm were deposited by co-evaporation of PbI_2_ and MAI. The vacuum chamber was evacuated to the same pressure of 1.6 × 10^–6^ mbar. The temperature of PbI_2_ and MAI were fixed at 300 °C and 180 °C. A pre-calibrated quartz crystal microbalance (QCM) sensor was used to monitor the rate of PbI_2_ and MAI and the thickness of MAPbI_3_. The deposition rate of PbI_2_ and MAI were 1 Ås^−1^ and 2.5 Ås^−1^, respectively.

### STXM measurements

STXM measurements were performed at the HERMES beamline of synchrotron SOLEIL. The details of the beamline are mentioned in Fig. 2 and elsewhere^44^. A Fresnel zone plate of 25 nm outer zone width (Applied Nanotools Inc., Canada) was used to focus the monochromatic X-rays to a spot size of (30 nm). The transmitted X-rays are detected by a photomultiplier tube (PMT) after being converted to visible photons by the phosphorous screen. The sample is raster scanned across the X-ray spot with the help of an interferometrically stabilized piezoelectric stage. The following protocol is recommended in order to perform studies in a reproducible fashion: (a) clean beamline optics is essential for investigating organic species, (b) if possible, in order to avoid contamination and degradation of the samples during transportation, a vacuum environment or isolated environment with minimum exposure to light is recommended. Spectral data presented in this communication (Figs. 3, 4 and 5) are primarily obtained from a stack of very low-resolution images measured at a range of predetermined energies. Each spectral point in these cases is obtained by integrating over each image. The energy range investigated for each sample is from 270 to 750 eV covering C K-edge, N K-edge, O K-edge, I M-edge, and Pb N-edge. To avoid any mechanical drift and focusing issues due to long range travel of the zone plate, we had split the scans into four partially overlapping sections from 270 to 390 eV, 370 to 450 eV, 430 to 570 eV and 550 to 750 eV. Focus corrections and beam alignment were performed between each chosen energy range. High spectral resolution (0.1 eV energy step) was used near the absorption edges of C, N, O, and I. In the intermediate energy ranges, an energy step of up to 5 eV was used. Before real measurement, a radiation damage study is recommended. In order to obtain spatially resolved spectra, energy stacks were obtained over an area of 2 × 2 microns by raster scanning the sample with a step size of 30 nm with the same energy definition as described above.

## Supplementary Information

Supplementary Information.

## Data Availability

The data generated during and/or analysed during the current study are available from the corresponding author on reasonable request.
