# Supplementary material for: Soft X-ray characterization of halide perovskite film by scanning transmission X-ray microscopy
Source: Sci Rep. 2022 Mar 16;12:4520. doi: 10.1038/s41598-022-08256-3 (PMC8927596; doi:10.1038/s41598-022-08256-3)
Supplement: Supplementary file 1 — Supplementary Information. [file 41598_2022_8256_MOESM1_ESM.docx]

Soft X-ray Characterization of Halide Perovskite Film by Scanning Transmission X-ray Microscopy

Haeyeon Jun^[a,b]^, Hee Ryung Lee^[a]^, Denis Tondelier^[a]^, Bernard Geffroy^[c,a]^, Philip Schulz^[d]^, Jean-Éric Bourée^[a]^, Yvan Bonnassieux^[a]^, and Sufal Swaraj^*[b]^

^[a]^LPICM, CNRS, Ecole Polytechnique, Institut Polytechnique de Paris, route de Saclay, 91128 Palaiseau, France

^[b]^Synchrotron SOLEIL, L'Orme des Merisiers Saint-Aubin, BP 48 91192 Gif-sur-Yvette Cedex, France

^[c]^Université Paris-Saclay, CEA, CNRS, NIMBE, LICSEN, 91191, Gif-sur-Yvette, France

^[d]^IPVF, 18 boulevard Thomas Gobert, 91120 Palaiseau, France

* Corresponding author

Phone number: +33 (0)1 69 35 81 82

E-mail address: [sufal.swaraj@synchrotron-soleil.fr](mailto:sufal.swaraj@synchrotron-soleil.fr)

Contents

Figure S1 presents energy-dispersive x-ray (EDX) spectra of methylammonium iodide (MAI) film on indium tin oxide coated glass. The experimental percentages of each element, including carbon, nitrogen, and iodine, are shown in Table S1. The atomic ratio of all components obtained by the measurement is different from the ratio in the chemical formula of CH_3_NH_3_I. The atomic percentage of iodide is much smaller than those of carbon and nitrogen indicating that most iodine is volatilize after deposition on sample substrate.


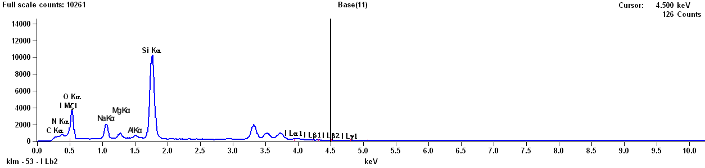


Figure S1 Energy-dispersive x-ray (EDX) spectra of methylammonium iodide (MAI) thin film as described in experimental details.

Table S1 Energy-dispersive x-ray spectroscopy (EDX) analysis of carbon (C), nitrogen (N), and iodine (I) in MAI film.

| **Element line** | **Net counts** | **Atom %** |
| --- | --- | --- |
| C K | 4217 | 10.82±1.2 |
| N K | 6305 | 20.43±1.8 |
| I L | 572 | 0.17±0.03 |

Surface morphologies of MAPbI_3_ with different deposition ratios of PbI_2_ and MAI are shown in Figure S2. In MAPbI_3_ film with the ratio of PbI_2_:MAI = 1:0.5, the average grain size is around 100 nm (Figure S2a). As the amount of MAI increases with the ratio PbI_2_:MAI = 1:1, the size of most grains does not change, however bigger grains with a size of 150 nm appear Figure S2b. When the ratio of PbI_2_ and MAI equals 1 to 2.5, the grain size is the biggest in all films, and the average grain size is around 250 nm Figure S2c. Figure S2d shows X-ray diffraction (XRD) patterns of MAPbI_3_ with the different ratios. Major peaks (2θ) at 14.2°, 28.6°, and 32.2° are shown in the spectra, and they indicate three tetragonal phases of (001), (220), and (301) in MAPbI_3_, respectively. In addition, the peak at 2θ = 12.5° (black star) is (001) phased PbI_2_. When the amount of MAI in deposition increases, the peak of PbI_2_ at 12.5° decreases, and it is negligible in MAPbI_3_ with the ratio of 1 to 2.5. Furthermore, the phase (110) in MAPbI_3_ is relatively more dominant compared than the phase (220) as the proportion of MAI increases. Table S2 indicates net counts and atomic percentages of each element (carbon, nitrogen, lead, and iodine) calculated from EDX spectra of MAPbI_3_ with different ratios of PbI_2_ and MAI. The atomic percentages of carbon and lead don’t change. However, the percentages of nitrogen and iodine increase as the amount of MAI increases.


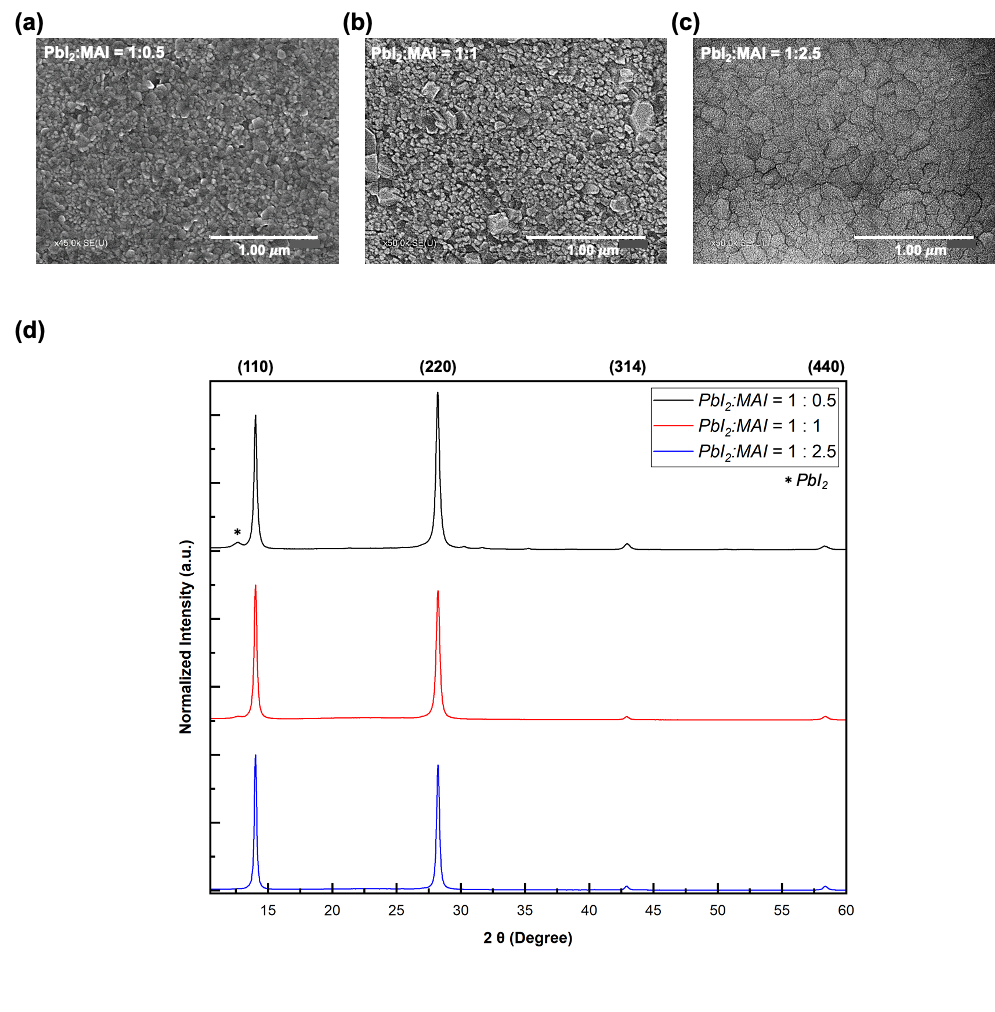


Figure S2 Scanning electron microscopy (SEM) images of MAPbI_3_ film with different deposition ratio of PbI_2_ and MAI a) 1:0.5, b) 1:1 and c) 1:2.5, d) X-ray diffraction (XRD) patterns of MAPbI_3_ film with different deposition ratio of PbI_2_ and MAI.

Table S2 Energy-dispersive x-ray spectroscopy (EDX) analysis of carbon (C), nitrogen (N), lead (Pb), and iodine (I) in MAPbI_3_ according to deposition ratio of PbI_2_ and MAI.

| PbI_2_: MAI deposition ratio | 1 : 0.5 | 1 : 1 | 1 : 2.5 |
| --- | --- | --- | --- |
| Net counts of C | 3613 | 4247 | 4550 |
| Atom % of C | 10.66 | 10.66 | 10.31 |
| Net counts of N | 4461 | 5431 | 6561 |
| Atom % of N | 18.49 | 19.07 | 21.19 |
| Net counts of Pb | 7411 | 9072 | 8290 |
| Atom % of Pb | 1.02 | 1.05 | 0.95 |
| Net counts of I | 5407 | 5982 | 6492 |
| Atom % of I | 2.04 | 2.19 | 2.27 |
| Pb/I ratio | 0.50 | 0.48 | 0.42 |
